# The effect of longstanding silicone oil on retina choroid and optic nerve in eyes with retinal detachment: an optical coherence tomography study

**DOI:** 10.1186/s12886-021-02239-0

**Published:** 2022-01-05

**Authors:** Umut Karaca, Murat Kucukevcilioglu, Ali Hakan Durukan, Dorukcan Akincioglu

**Affiliations:** 1 Department of Ophthalmology, Isparta Suleyman Demirel University Faculty of Medicine, Isparta, Turkey; 2 Department of Ophthalmology, University of Health Sciences, Gulhane School of Medicine, Ankara, Turkey; 3 Department of Ophthalmology, Ataturk City Hospital, Antalya, Turkey

**Keywords:** Choroid, Long-term effects, Optical coherence tomography, Retinal layers, Silicone oils

## Abstract

**Background:**

The study aims to evaluate peripapillary retinal nerve fiber layer thickness (RNFL-T), central macular thickness (MT), choroidal thickness (CT), and thickness of each retinal layer after automatic segmentation in patients who underwent retinal detachment (RD) repair with longstanding silicone oil tamponade.

**Methods:**

We enrolled 33 patients who underwent complicated primary rhegmatogenous RD surgery and followed up with a long-term silicone tamponade were included in this retrospective comparative (case–control) study. RNFL-T, CT, and thickness of each retinal layer after automatic segmentation analysis were measured after the longstanding silicone removal surgery.

**Results:**

The mean silicone oil removal time was 15.1 ± 15.2 (7–70) months. The overall average thickness of the RNFL was 90.7 ± 13.6 μm in the operated eyes and 118.3 ± 35.6 μm in the sound eyes, with a statistically significant difference. The overall average central MT was 186.3 ± 57.7 μm and was significantly lower in the operated eyes than in the sound eyes. Inner retinal layers of the study group showed a significant thinning in the nerve fiber layer, ganglion cell layer, inner plexiform layer, and inner nuclear layer as compared to that of the sound eyes. The subfoveal CT was 213.7 ± 86.6 μm in the study eyes and 217.7 ± 115.5 μm in the control eyes. There was no significant difference between the study eyes and controls.

**Conclusion:**

The effects of silicone oil on the retina remain uncertain; however, morphological results in our study have shown direct or indirect silicone oil–induced toxicity, especially in the inner retinal layers.

## Backround

Silicone oil is an ocular endotamponade that is used in vitreoretinal surgery to sustain retinal attachment after the repair of complex retinal detachments (RDs), giant retinal tears, proliferative vitreoretinopathy (PVR), trauma, and endophthalmitis. Its high viscosity and surface tension properties assist in providing an excellent structural support as compared to the intraocular gas tamponade in selected cases. Because silicone oil causes, especially, anterior segment complications in terms of retention time in the eye, it should be removed when it is no longer needed as an intraocular tamponade [1, 2]. Another crucial issue is the effect of silicone oil on the posterior segment and retina. There are many studies in the literature that investigated the loss of vision and tried to reveal possible causes, especially after the use of silicone oil. Potential blameworthy mechanisms include optic nerve damage due to direct tissue infiltration or/and thinning in the inner retinal layers because of horizontal bipolar cell damage [3, 4].

Spectral domain optical coherence tomography (SD–OCT) technology helps us in identifying pathologic changes in the retinal layers, optic disk, and even choroidea. In SD-OCT, macular thickness varies depending on age, gender and spherical refraction, and individual variations can be observed. However, there is high congruence between both eyes of the same individual [5].

The purpose of this study is to evaluate peripapillary retinal nerve fiber layer thickness (RNFL-T), central macular thickness (MT), choroidal thickness (CT), and thickness of each retinal layer after automatic segmentation in patients who underwent RD repair with longstanding silicone oil tamponade.

## Materials and methods

### Participants

We enrolled 33 patients who underwent complicated rhegmatogenous RD surgery with silicone oil tamponade between January 2015 and December 2019 in this retrospective comparative (case–control) study. Patients whose retina was detached because of PVR and followed up with a long-term silicone tamponade were included in the study. Exclusion criteria included a history of glaucoma, a history of ocular diseases that might affect SD–OCT imaging (cataract, corneal opacity, age-related macular degeneration, degenerative myopia, coloboma, etc.), a history of systemic diseases that might affect retinal/choroidal blood flow, a history of medication that might affect retinal tissues, and uncomplicated primary detachment surgeries where silicone oil is removed within 6 months. The healthy other eyes were selected as controls.

### Surgical technique

All surgical procedures such as pars plana vitrectomy and silicone oil tamponade were performed under retrobulbar anesthesia by one of two surgeons (AHD and MK). The surgical technique included the removal of PVR, intraretinal and subretinal fibrotic materials, sealing of tears with endolaser photocoagulation, and injection of silicone oil. The retina was reattached successfully either with the support of perfluorocarbon liquid (Okta-line, Bausch & Lomb) or through direct air–silicone oil exchange (SIL1000-SIL5000, DORC, Zuidland, The Netherlands) in all the cases. Importantly, cataract surgery was performed peroperatively in the phakic patients. Silicone extraction was performed at the earliest after the seventh month of surgery. After silicone extraction, the patients underwent a full ophthalmoscopic examination in the first week, the first month, and every two months after. SD–OCT and enhanced depth imaging (EDI–OCT) scanning were performed at each visit.

### OCT measurements

**Fig. 1:**
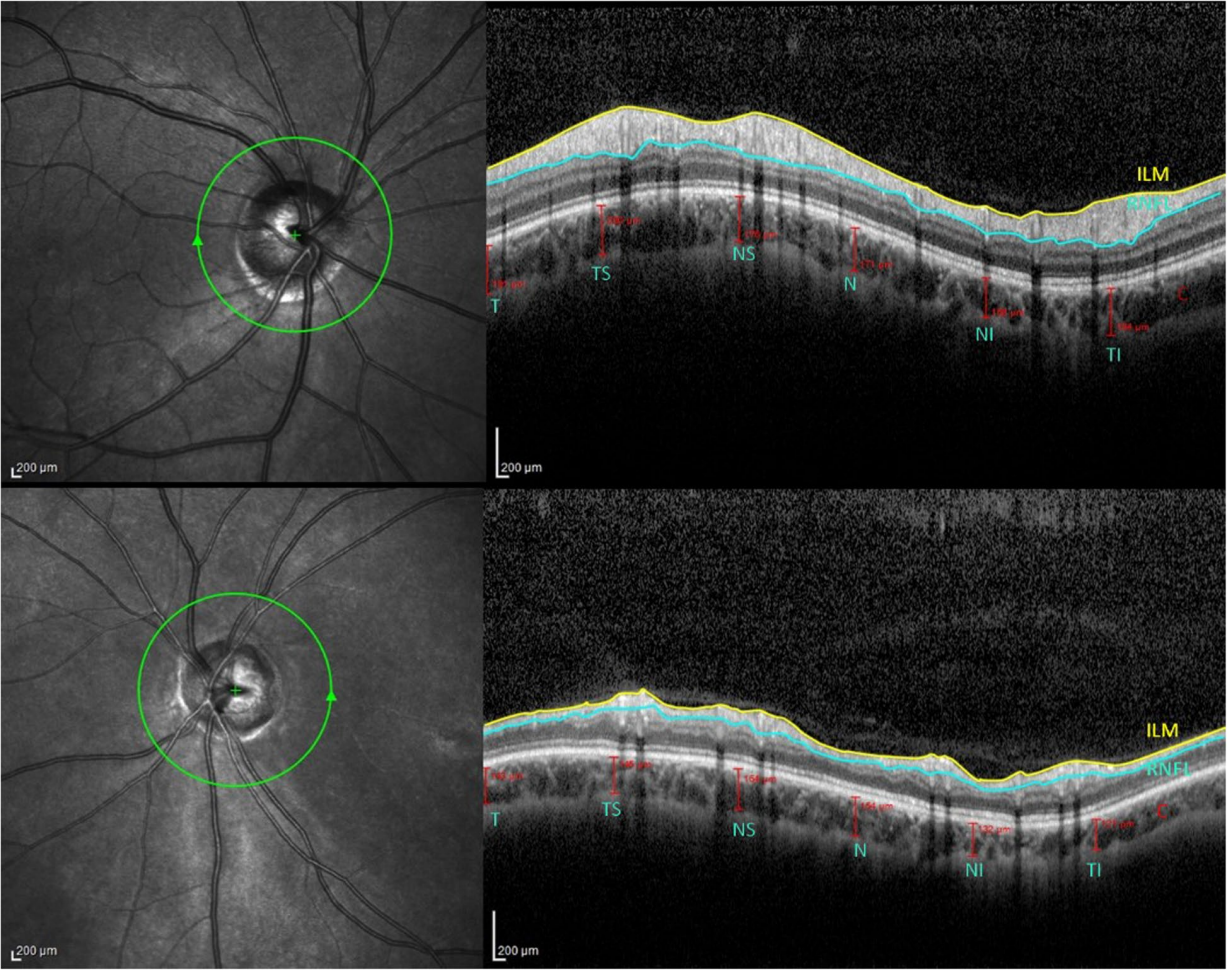
Peripapillary choroidal thickness measurements. (ILM, internal limiting membrane; RNFL, retinal nerve fiber layer; T, Temporal; TS; Superotemporal, TI; Inferotemporal N, Nasal; NS, Superonasal; NI, Inferonasal)

RNFL-T measurements were obtained by performing 360° peripapillary circle scans with a diameter of 3.4 mm in an SD–OCT device (Spectralis, Heidelberg Engineering, Heidelberg, Germany). Submacular and peripapillary choroidal measurements were performed by an experienced technician in the afternoon (regarding the diurnal variation of choroidal blood flow) under the normal room illumination with the EDI–OCT mode of the same device. Although RNFL-T was automatically measured by OCT, CT measurements were performed manually by two graders in the central macula and six regions of peripapillary choroid. CT was manually determined as the outer surface of the hyperreflective line corresponding to the retinal pigment epithelium and the inner surface of the sclera (Fig. 1). Two ophthalmologists (MK and UK) measured CT at the fovea and all quadrants in the peripapillary region. A difference of only 10 μm (micron) between two measurements was determined as an acceptable criterion. If this limit value was exceeded, then a third ophthalmologist took the measurement and an average of three measurements was taken.

**Fig. 2:**
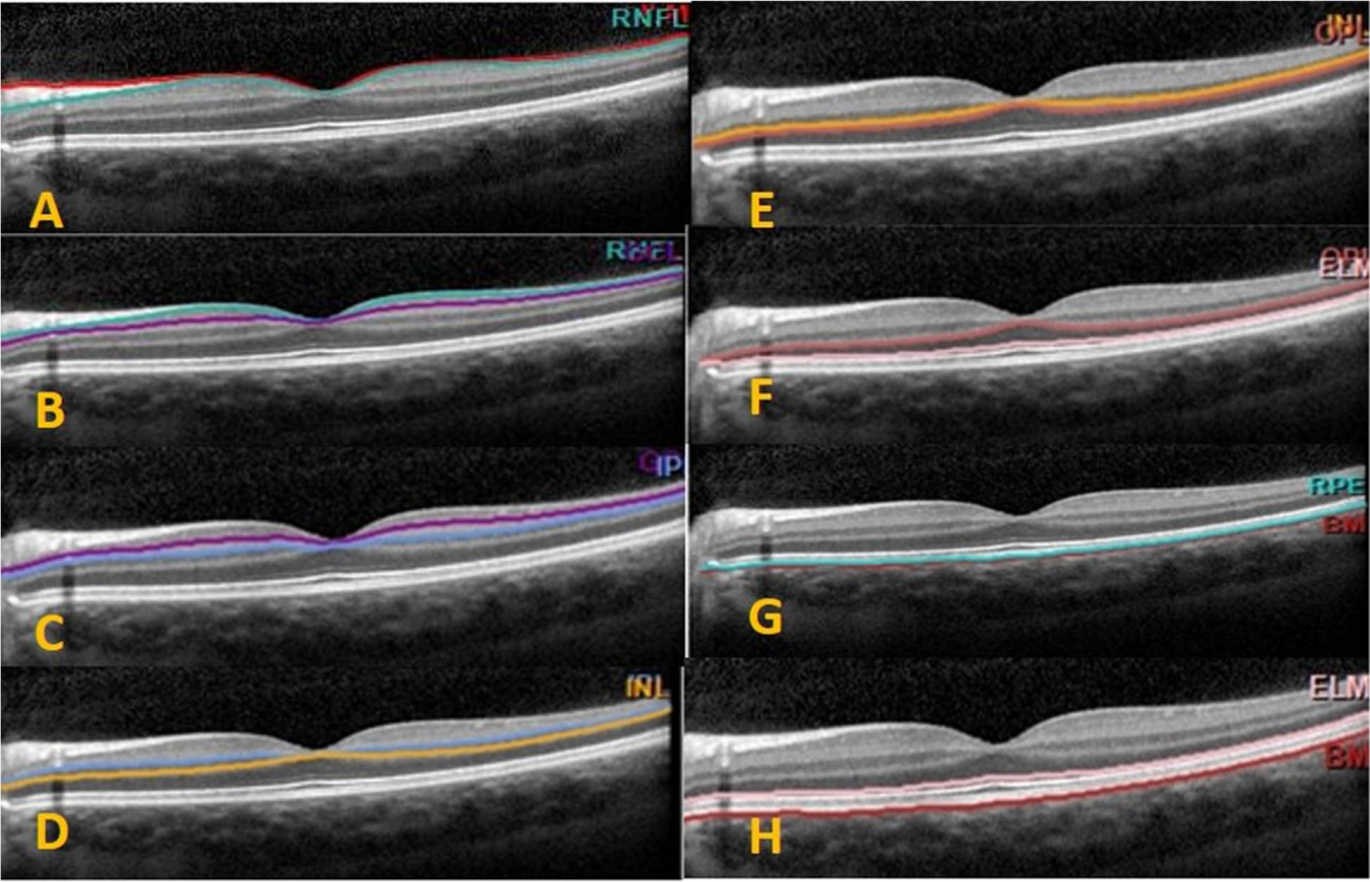
A representative figure of retinal layer division determined by the automated segmentation application of the Spectralis OCT. The segmentation software automatically marked the ten retinal layers (**A**: RNFL, retinal nerve fiber layer; ILM, internal limiting membrane; **B**: GCL, Ganglion cell layer; **C**: IPL, inner plexiform layer; **D**: INL, inner nuclear layer; **E**: OPL, Outer plexiform layer; **F**: ONL: Outer nuclear layer; **G**: PR, photoreceptors; RPE, retinal pigment epithelium; H: BM, Bruch membrane; ELM, external limiting membrane)

Automated retinal segmentation of SD–OCT retinal images was applied to distinguish each retinal layer and quantify its thickness (Segmentation Technology; Heidelberg Engineering, Germany). The retina was automatically segmented into ten layers and the mean thickness of each layer was particularly calculated. The degree of change in thickness of each retinal layer within 1 mm of ETDRS subfields was analyzed for each patient (Fig. 2).

### Statistical analysis

Data analysis was performed with SPSS Statistics 20.0 software package. The normality of data was evaluated using the Kolmogorov–Smirnov test. Non-parametric tests were used because of the small sample size. A *p*-value of 0.05 or less was considered significant for this study.

## Results

Table 1 summarizes the demographic and baseline characteristics of the participants. The mean age of the patients was 62.05 ± 15.7 (20–89) years; 14 of which were women (42.4%). The mean silicone oil removal time was 15.1 ± 15.2 (7–70) months. Additionally, the mean visual acuity of the patients was 1.71 ± 0.96 (LogMar) before silicone removal surgery, whereas the mean final visual acuity was 1.61 ± 0.95 (LogMar). The patients were observed to be normotensive with or without medication in the postoperative period.

**Table 1:** Demographic and baseline characteristics of the subjects (SD: Standard deviation)

Characteristics	Patients
**Num. of eyes/patients**	33
**Age (Mean ± SD), years**	62.05 ± 15.7 (20–89)
**Gender (female/male)**	14/19
**Lens (Phakic/Pseudophakic)**	10/23
**Silicone type (1000CS/5000 CS)**	25/8
**Silicone removal time (Mean ± SD), months**	15.1 ± 15.2 (7–70)
**Visual Acuity (Initial) (LOGMAR)**	1.71 ± 0.96
**Visual Acuity (Final) (LOGMAR)**	1.61 ± 0.95

### OCT measurements

The overall average thickness of the RNFL was 90.7 ± 13.6 μm in the operated eyes and 118.3 ± 35.6 μm in the sound eyes, with a statistically significant difference (*p* < 0.05). The overall average central MT was 186.3 ± 57.7 μm and was significantly lower in the operated eyes as compared to the sound eyes (*p* < 0.05). The subfoveal CT was 213.7 ± 86.6 μm in the study eyes and 217.7 ± 115.5 μm in the control eyes. There was no significant difference between the study eyes and controls (*p* > 0.05).

Table 2 summarizes average RNFL-T and CT values at different peripapillary locations. There was no significant difference between the study eyes and controls regarding the RNFL of the peripapillary zones except in the temporal zone. There was no significant difference between the study eyes and controls regarding the CT of the peripapillary zones. The RNFL and CT measurements were not significantly correlated for any peripapillary location (|r| ≤ 0.17, *p* > 0.05). Although there was no significant difference in the peripapillary CT, the overall average CT was lower in the silicone-filled eyes.

**Table 2:** Average RNFL and choroidal thickness values at different peripapillary locations (RNFL: Retinal Nerve Fiber Layer, CT: Choroidal Thickness)

	RNFL			CT		
	Study eye	Control	*P*-value	Study eye	Control	*P*-value
**Nasal**	76.4 ± 12.4	86.3 ± 35.8	0.28	150.3 ± 63.0	167.5 ± 72.9	0.20
**Inferonasal**	101.3 ± 22.3	116.2 ± 50.6	0.22	141.7 ± 87.5	177.3 ± 85.1	0.17
**Inferotemporal**	110.8 ± 47.3	123.7 ± 32.2	0.10	159.9 ± 68.2	182.6 ± 97.4	0.17
**Temporal**	67.5 ± 14.4	92.8 ± 37.0	0.03*	159.8 ± 59.5	176.9 ± 81.6	0.15
**Superotemporal**	111.8 ± 42.5	129.9 ± 37.1	0.08	142.9 ± 64.9	164.5 ± 84.7	0.17
**Superonasal**	99.6 ± 30.4	99.7 ± 42.0	0.99	121.0 ± 59.2	155.7 ± 86.6	0.06

### Retinal segmentation analysis

Table 3 presents the difference in the thickness of each retinal layer within 1 mm of ETDRS subfield in the long-term silicone-filled eyes and the sound eyes. Inner retinal layers of the study group showed a significant thinning in the nerve fiber layer, ganglion cell layer (GCL), inner plexiform layer (IPL), and inner nuclear layer (INL) as compared to that in the sound eyes. Although not statistically significant, thinning was also determined in the outer retinal layers. Spearman’s correlation analysis was applied to determine the factors related to segmental retinal thinning. There was no correlation between retinal layer thinning and age and gender (*p* > 0.05). Silicone retention time has a strong negative correlation with RNFL thinning at almost all quadrants. Correlations between postoperative RNFL thinning and, silicone retention time was summarized in Table 4. There was solely a positive correlation between INL thinning and final visual acuity (*r* = 0.679, *p* < 0.05).

**Table 3:** The thickness of retinal layers after segmentation

	Study eye	Control	***P*** value
**Nerve Fiber Layer (mean, microns)**	11.5 ± 2.1	24.1 ± 4.0	0.01*
**Ganglion Cell Layer (mean. microns)**	18.7 ± 3.2	37.0 ± 3.4	0.01*
**Inner Plexiform Layer (mean. microns)**	23.2 ± 2.6	40.2 ± 3.2	0.01*
**Inner Nuclear Layer (mean. microns)**	24.9 ± 3.1	40.6 ± 2.9	0.01*
**Outer Plexiform Layer (mean. microns)**	31.3 ± 3.4	35.5 ± 2.5	0.08
**Outer Nuclear Layer (mean. microns)**	80.7 ± 7.7	83.3 ± 6.5	0.35
**Pigment Epithelium (mean. microns)**	16.6 ± 2.1	18.1 ± 1.7	0.08

**Table 4:** Correlations between postoperative RNFL thinning and age, gender,silicone retention time

	Age		Gender		Silicone retention time	
	*r*	*p*	*r*	*p*	*r*	*p*
**RNFL- NASAL**	-,155	,55	,072	,78	-,676	,003*
**RNFL-INFERONASAL**	,020	,94	-,048	,85	-,512	,03*
**RNFL-INFEROTEMPORAL**	-,097	,71	,108	,67	-,284	,27
**RNFL-TEMPORAL**	,229	,37	-,433	,08	-,055	,83
**RNFL-SOPEROTEMPORAL**	-,206	,42	,361	,15	-,666	,004*
**RNFL-SUPERONASAL**	,253	,32	-,144	,58	-,645	,005*

## Discussion

In this study, we investigated the effects of long-term silicone oil on the central retina, optic disk, and choroid of the study eyes as compared to that of the sound eye by OCT imaging. The study results point that long-term silicone oil retention in the eye greatly affects the inner retinal layers. The statistically significant thinning in the temporal RNFL of study eyes as compared to that of control eyes further supports this finding. Additionally, the absence of a statistically significant difference in the CTs when compared to the control eyes suggests that the nutrition of the outer layers of the retina and optic disk may not be compromised because of silicone oil. Another remarkable finding in our study was the strong positive correlation between INL thinning and final visual acuity. Recent studies have demonstrated the variability of MT and RNFL-T in healthy individuals; therefore, we assumed that it would be more appropriate to use the other healthy eye of the same individual as controls [6].

Silicone oil is preferred as a long-term endotamponade that enhances the success rate of detachment repair and anatomical recovery with its hydrophobic nature. It has been selected especially for retinal detachments with giant tears and with ≥ group B PVRs [7]. Emulsification of silicone oil is the main reason that promotes the most common complications in the anterior and posterior segments of the eye. The amount of time silicone oil remains in the eye is the most crucial factor in the development of emulsification and so in reducing its complications [8]. The effects of long standing silicone oil on the retina has not been sufficiently investigated yet.

The effects of silicone oil on the retina and the causes of vision loss after removal have been previously investigated in several studies. Although animal studies have reported discordant results, first clinical OCT studies about morphological changes after successful macula-on or macula-off detachment surgeries focused on the alterations of outer retinal layers associated with poor visual outcomes [9–15]. Former OCT studies highlighted that the central foveal thickness alterations due to silicone oil tamponade was correlated with the final vision acuity level [14, 15]. Christensen and la Cour reported severe visual loss associated with significant retinal (subfoveal) thinning after the use of silicone oil in 33% of patients who underwent macula-on RD. [13] Bolukbasi et al. reported statistically significant thinning in the subfoveal choroidal thickness; and Delolme et al. presented outer retinal layer changes with the successful repair of rhegmatogenous RD. [12, 16] Although alterations of the photoreceptor outer segments and changes in the IS/OS band had been previously accused, later studies suggested that the thinning of inner retinal layers and ganglion cell loss may be the potential reasons of vision loss [17, 18].

Although ganglion cells gradually decrease with age, silicone oil is blamed for the pathological death of retinal ganglion cells [19]. Lee et al. have found that retinal thickness, GCL, outer plexiform layer, and outer nuclear layer thicknesses were significantly thinner in the silicone oil group with a mean duration time of 101 days when compared with gas-filled eyes [18]. Caramoy et al. reported reduced ganglion cell and IPL even in short-term silicone oil–based endotamponade [20]. The potential mechanisms blamed for significant thinning were inflammatory process due to hyper reactivity toward silicone oil/emulsified silicone oil, which resulted in apoptotic cytokine discharge, dysfunction of Muller cells, and retinal toxicity [21–23]. All these previous studies have been performed in primary rhegmatogenous RDs or uncomplicated vitreoretinal surgeries. On the contrary, our study focused on complicated surgeries with long-term silicone oil tamponade. However, to date, we have not seen a study that investigated silicone oil–related changes in eyes with such a long period of retention time.

In complicated RD surgeries, anatomical success was defined as retinal reattachment and functional success was defined as the achievement of vision better than 5/200 [24]. Although the retinal reattachment can be achieved at the end of complicated vitreoretinal surgeries, its functional results were unsatisfactory [25, 26]. The reason of this discordance is exactly unknown; however, the severity of underlying diseases and prolonged silicone oil tamponade may be the etiological factors. Although there is no study showing the effects of long-term silicone oil tamponade, Scott et al. showed a significant correlation between an early removal of silicone oil and an improved visual acuity [25]. Our study is the first one that investigates the retinal and choroidal morphological differences in eyes with longstanding silicone oil tamponade. Our study showed that there was no change in the CT as compared to the other eye even in cases where silicone oil remained for a long time. Moreover, we found that silicone oil mainly affected the inner retinal layers, and there was significant thinning of GCL, INL, and RNFL. In our study, the main reason why silicone oil remained in the eye for a longer time is that the patient did not comply with the surgery program and did not come to follow-up visits. However, it will be critical to remove the silicone oil tamponade as early as possible to minimize the thinning effect, especially on the inner retinal layers.

The main question to be asked for this study is whether the retinal changes revealed are due to complicated RD or long-term silicone oil retention. (over 6 months). The main retinal region where pathological changes are observed after RD are the photoreceptor sequence and outer retinal layers [27]. Additionally, the effect of silicone oil on the retina is related to the time it stays in the eye rather than its physical properties. Long-term tamponade with silicone oil for more than 9 months causes an increase in the arteriovenous flow difference and narrowing of the retinal arterioles [28].

Limitations of our study are its retrospective nature, small sample size, and nonrandomized patients. We included mostly macula-off recurrent RDs, some of which needed perfluorodecalin for reattachment and some needed cataract removal at the same session. Different types of silicone were used and the choice of silicone oil was solely determined by the surgeon at the time of surgery. Multiple surgeries, previous solved macular complications like epiretinal membranes, and probable undefined emulsification of silicone oil weakened the standardization.

The effects of silicone oil on the retina remain uncertain, but morphological results in our study have shown direct or indirect silicone oil–induced toxicity, especially in the inner retinal layers. Prospective long-term studies with a large sample size are needed to confirm our observations.

## Data Availability

The datasets used and/or analyzed during the current study available from the corresponding author on reasonable request. Unfortunately, the data is not publicly available due to local data protection laws.
